# Forecasting of Flash Floods Peak Flow for Environmental Hazards and Water Harvesting in Desert Area of El-Qaa Plain, Sinai

**DOI:** 10.3390/ijerph19106049

**Published:** 2022-05-16

**Authors:** Ismail Abd-Elaty, Hanan Shoshah, Martina Zeleňáková, Nand Lal Kushwaha, Osama W. El-Dean

**Affiliations:** 1 Department of Water and Water Structures Engineering, Faculty of Engineering, Zagazig University, Zagazig 44519, Egypt; eng_abdelaty2006@yahoo.com (I.A.-E.); hananshoshah82@gmail.com (H.S.); maria.hlinkova13@gmail.com (O.W.E.-D.); 2 Institute of Environmental Engineering, Faculty of Civil Engineering, Technical University of Košice, 04200 Košice, Slovakia; 3 Division of Agricultural Engineering, ICAR-Indian Agricultural Research Institute, New Delhi 110012, India; nand.kushwaha@icar.gov.in

**Keywords:** watershed, forecast, El-Qaa Plain, Sinai and WMS, hazards, hydrograph

## Abstract

Water resources in arid and semi-arid regions are limited where the demands of agriculture, drinking and industry are increasing, especially in drought areas. These regions are subjected to climate changes (CC) that affect the watershed duration and water supplies. Estimations of flash flooding (FF) volume and discharge are required for future development to meet the water demands in these water scarcity regions. Moreover, FF in hot deserts is characterized by low duration, high velocity and peak discharge with a large volume of sediment. Today, the trends of flash flooding due to CC have become very dangerous and affect water harvesting volume and human life due to flooding hazards. The current study forecasts the peak discharges and volumes in the desert of El-Qaa plain in Southwestern Sinai, Egypt, for drought and wet seasons by studying the influence of recurrence intervals for 2, 5, 10, 25, 50 and 100 years. Watershed modeling system software (WMS) is used and applied for the current study area delineation. The results show that the predictions of peak discharges reached 0, 0.44, 45.72, 195.45, 365.91 and 575.30 cubic meters per s (m^3^ s^−1^) while the volumes reached 0, 23, 149.80, 2,896,241.40, 12,664,963.80 and 36,681,492.60 cubic meters (m^3^) for 2, 5, 10, 25, 50 and 100 years, respectively, which are precipitation depths of 15.20, 35.30, 50.60, 70.70, 85.90 and 101 mm, respectively. Additionally, the average annual precipitation reached 13.37 mm, with peak flow and volume reaching 0 m^3^ s^−1^ where all of water harvesting returned losses. Moreover, future charts and equations were developed to estimate the peak flow and volume, which are useful for future rainwater harvesting and the design of protection against flooding hazards in drought regions due to CC for dry and wet seasons. This study provides relevant information for hazard and risk assessment for FF in hot desert regions. The study recommends investigating the impact of recurrence intervals on sediment transport in these regions.

## 1. Introduction

Flash flooding is becoming more common as a result of extreme weather conditions, and affects on human and animal mortality, accidents, mental health difficulties, vector-borne infections, and waterborne diseases [1,2]. Many coastal areas are effected by freshwater boundary changes due to over-pumping and CC [3]. FF hazards are responsible for economic and human life loss. Estimation of runoff is an important hydrological aspect and plays a vital role in the planning and management of natural processes, such as soil erosion, flood and drought risks. The limitation is the availability of hydrological data in dry valleys [4]. The variation in high-velocity of the rainfall over a short duration is responsible for FF values the heavy sediment load that threatens the lower part of settlements in the wadis and affects the livelihood of the community of the watershed [5]. 

Rainfall forecasting is critical for providing early warnings before FF events, allowing disasters to be avoided or minimized [6]. The volume of water collected, penetrated, stored, evaporated, transpired and subtracted from the precipitation (i.e., runoff) is calculated using the rainfall-runoff model. Infiltration and evaporation are two types of flood losses. Infiltration is calculated using the Green–Ampt model (GAM), the Horton formula, and the soil conservation service (SCS) curve number (CN) approach [7]. The findings of the two approaches, i.e., the GAM and CN equation, were compared by Smemoe et al. (2004) [8]. The results showed that the GAM was superior to the CN method. Chahinian et al. (2005) [9] applied Philip, Morel-Seytoux, Horton and SCS infiltration models to the test on 14 different events. The mathematical framework and calibration parameters of these models were different, but the input hydrologic data were the same. The results revealed that the Morel-Seytoux model outperformed the others, with the SCS coming in last. Horton’s model performed better than Philip’s in terms of overall consistency.

The runoff discharge could be estimated by several synthetic unit hydrograph (UH) methods including SCS dimensionless UH and Snyder UH, while the peak discharge could be estimated by the rational approach. Jena and Tiwari (2006) [10] employed GIS to investigate two watersheds and associated sub-watersheds in West Bengal’s Midnapore and Bankura districts. Flow data and UH were used to create the runoff hydrographs. Garambois et al. (2014) [11] used statistical analysis to investigate FF storms and the hydrological responses of catchments in the Pyrenean foothills up to the Aude area. The findings showed that increasing initial soil saturation led to faster catchment flood response times, ranging from 3 to 10 h, as well as flooding caused by rainfall near the catchment outlet, where the topography was lower.

Due to severe weather conditions, mostly heavy rainfall, the Sinai Peninsula receives a great deal of rain, which generates a lot of FF in the area. The impact of floods in south Sinai has increased in recent decades, and several researchers, including JICA (1999) [12], Youssef et al. (2011) [13], Nahla (2016) [14] and Maria et al. (2020) [15] have evaluated its impact values. The main cause of FF in the Sinai Peninsula is a short duration of rainfall accompanied by snowmelt runoff and a low infiltration capacity of the soil, among other factors, resulting in an increased overland flow even though total fall rainfall amounts in these areas are relatively small [16]. Awadallah et al. (2011) [17] developed the Intensity Duration Frequency (IDF) of a region in Angola’s north-west, using limited data from ground rainfall stations and TRMM data. Cools et al. (2012) [18] used the best available data to construct and assess an early warning system (EWS) for FF in Egypt’s Sinai Peninsula. According to the data, 90 percent of the entire rainfall volume was lost due to infiltration and transmission losses. Wahid et al. (2016) [19] used GIS to analyze the developed datasets for runoff and potential flash floods, as well as to visualize the spatial distribution of flood and runoff potential in the southwestern Sinai coastal plain. The study concluded that the slope and soil types are the two most important elements in determining runoff levels and FF potential.

According to the Intergovernmental Panel on CC (IPCC, 2013, 2014) [20,21] a significant variations for the regional temperature and precipitation from the global-scale pattern are projected (Christensen et al. 2013) [22]. Climate data from 1970 to 2014 revealed rapid CC in Egypt’s Sinai Peninsula, with decreased rainfall and rising average temperatures. For several years, this tendency resulted in severe droughts that were abruptly interrupted by high and erratic rainfall that varied greatly in locations and duration. Many plant and animal species’ population dynamics will also be badly impacted, with many of them being essential to residents [23]. The El-Qaa Plain in the Sinai Peninsula is a region that is constantly growing in population [24]. Mostafa et al. (2019) [25] estimated the future temperature and precipitation trends in Egypt for 2100 based on the past data from 1950 to 2017; the results showed that, in Northern Egypt, the projected patterns of annual precipitation have been reduction to 0.48 from 1.40 mm yr^−1^.

The current study was simulated using the Watershed Modeling System (WMS) to forecast the peak flooding in the desert of El-Qaa plain in Southwestern Sinai, Egypt, due to the impact of CC which affect the recurrence intervals for 2, 5, 10, 25, 50, and 100 years. The prediction of future hydrographs for peak discharge and volume will be estimated in the current study with the future recurrence intervals and rainfall depths. Also, the estimation of future flood volumes and depths will help in the development of this desert area which consider the only water resource for its people.

## 2. Materials and Methods

### 2.1. Wadi El-Aawag Watershed (Case Study Area)

**Figure 1:**
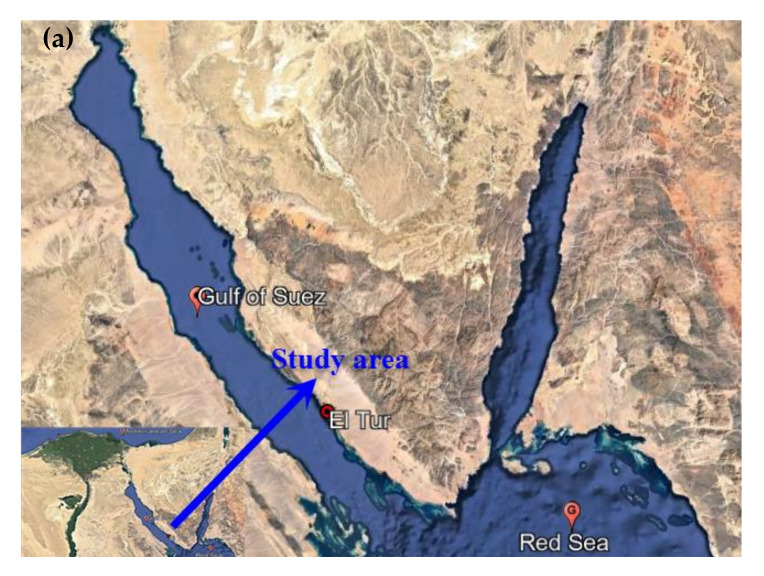

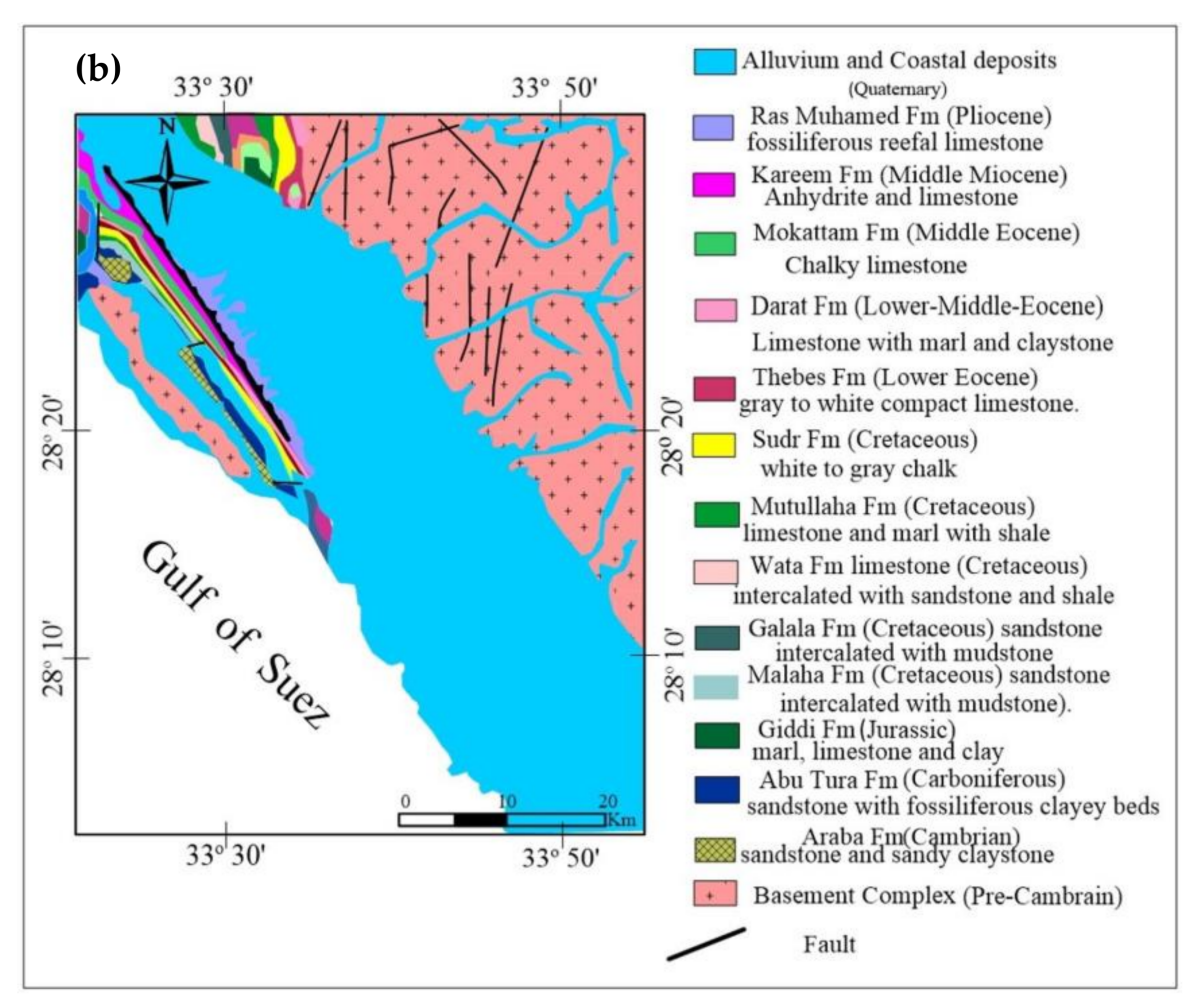
El-Aawag Watershed for (**a**) location map of the study area (Google earth 2022) and (**b**) geological map. (after, UNSECO 2004 [30]).

The southwestern corner of the Sinai Peninsula contains the El-Qaa Plain, located between latitudes 28°15′, 28°45′ north and longitudes 33°20′, 34°00′ east and neighbouring the Gulf of Suez [26]. The watershed of Wadi El-Aawag (WEA) is considered one of the largest basins in the Gulf of Suez’s drainage system with an area of about 1960 km^2^. The watershed direction extends generally from north and northeast to southwest for about 58 km; the location of the current study is presented in Figure 1a. In addition, it debouches to the Gulf of Suez coastal plain, which is locally named Sahl El-Qaa. This plain stretches for around 3500 km^2^ along the southwest coast of Sinai. A test site was chosen in the Sinai Peninsula’s El-Qaa Plain as it falls under promising development zones in the Sinai Peninsula, particularly in terms of tourism. These opportunities have already resulted in a progressive growth in the number of people living in the area, as well as an extension of land exploitation [27,28].

WEA watershed’s geology is predominantly composed of Precambrian and Cambrian rocks. The Quaternary deposits (wadi deposits and undifferentiated deposits) occupy mainly the Sahl El-Qaa area (El-Qaa Plain), which is a promising area for groundwater reserves (see Figure 1b).

The hydrogeology of Sahl El-Qaa is drained by several watersheds (wadies), which originate from these granitic mountainous masses. These wadies are buried through different cycles of sedimentation and alluviation during the Quaternary times and become active during the rainy periods [29]. These various drainage networks play an important role in the water supply in the studied area.

### 2.2. Meteorological Data and Hydrological Frequency Analysis

The historical climate data are taken from the weather of El Tur station. The recorded Tmin, annual average temperature (°C); Tmax, annual average maximum temperature (°C); Taver, annual average; V, annual average wind speed (km hr^−1^); P_T_, total annual precipitation of snow (mm); RA, number of days with rain (-); Pmax, maximum daily rain or snow precipitation total annual (mm); and Paver, average annual rain or snow precipitation total annual (mm), are used in the current study. 

Table 1 presents the annual rainfall at El Tur station from 1995 to 2021, with the maximum value reaching 70.10 mm in a day and 273.31 mm in a year. The data show that the trends of precipitation are variable in that it was increased in 2015 and 2017, also it was decreased from 2018 to 2020 and increased in 2021, which will affect the estimation of recurrence intervals and peak floods.

**Figure 2:**
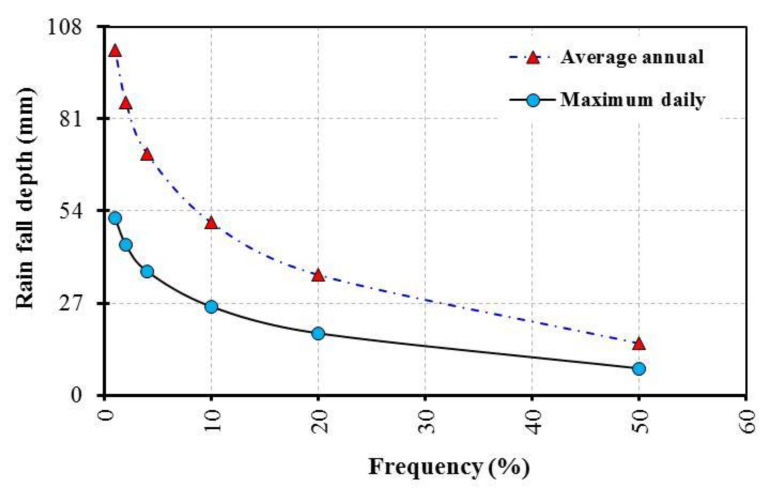
Relation between rainfall depth and frequency for El Tor station.

The predicted values of precipitation for different recurrence intervals were estimated using the hydrological frequency analysis (hyfran-plus) software version-v2.1, which is available at this link (http://www.wrpllc.com/books/HyfranPlus/indexhyfranplus3.html accessed on 11 April 2022) for different distributions including exponential, GEV, Gumbel, Weibull, Normal, Gamma and Log-Pearson type III. The data were carried out by the best fitting using the exponential distribution, as shown in Figure 2. The predicted values of precipitation are 11, 26.40, 38.10, 53.50, 65.10, 76.80 and 88.40 mm for 2, 5, 10, 25, 50, 100 and 200 years. The following equation was used to estimate the rainfall frequency to reach 50%, 20%, 10%, 5%, 4%, 2% and 1% for the recurrence intervals of 2, 5, 10, 25, 50 and 100 years.

The following equation was used to calculate the instantaneous rainfall intensity at any point during the storm duration:

(1) $$I_{t}=(\frac{60\times P_{t}}{t})$$

where *I_t_* = instantaneous rainfall intensity at time (*t*) from the storm start (mm h^−1^); *P_t_* is the precipitation depth recorded at time (*t*) from the storm start (mm); and *t* is time passed from the storm start (min).

### 2.3. Watershed Modeling

**Figure 3:**
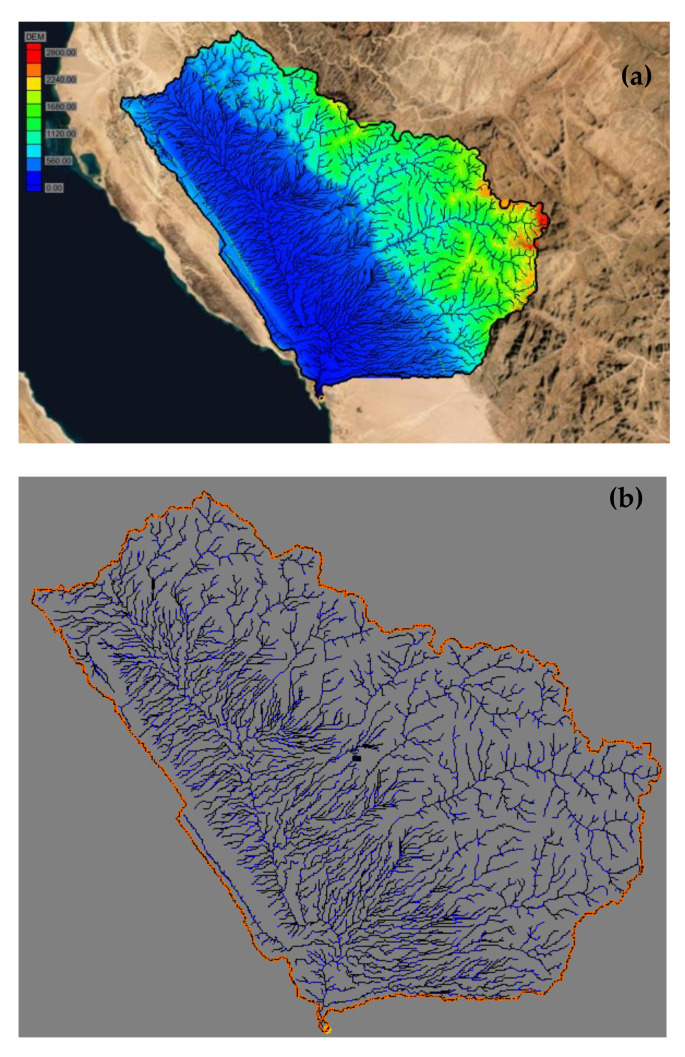
Study area (**a**) digital elevation model and (**b**) watershed characteristics in the study area.

The watershed modeling system (WMS) (https://www.aquaveo.com/downloads.wms accessed on 11 April 2022) was applied for the current study to delineate the stream flow and discharges based on the key geologic features and rock contacts. The model of the WEA watershed attains the 6th order (see Figure 3).

The drainage network of the study area was delineated using the WMS from Digital Elevation Model (DEM) with 30 m resolution (http://gdem.ersdac.jspacesystems.or.jp/search.JSP accessed on 11 April 2022). The losses for the watershed in the WMS were estimated using the SCS Curve Number (CN) method (Soil Conservation Service, 1972) [7]; this method considering the watershed for soil type, land cover and antecedent moisture condition.

SCS (1972) [7] developed the dimensionless UH method, which consists of the lag time (*T_L_*) in hr. The peak discharge was estimated using HEC-1 in the WMS based on UH, using the SCS method. The rainfall for the storm distribution was generated using the SCS type II which is applicable to rainfall stations where extreme storms last more than 3 hrs on average, and its distribution is suitable for deriving the 24-h time distribution during extreme events in many regions [17,31]. The WMS uses the time of concentration to estimate the lag time and compute peak flow and time to peak from the following equations used in the calculation of the main flood factors [32]:

(2) $$T_{c}=\frac{0.00013L^{0.77}}{S^{0.385}}$$

(3) $$T_{L}=0.60T_{c}$$

(4) $$T_{p}=\frac{T_{r}}{2}+T_{L}$$

(5) $$Q_{p}=\frac{2.08\times A}{T_{p}}$$

where L is length of the overland flow in feet; S is average overland slope in ft/ft (-); *T_c_* is the time of concentration in hours; *T_r_* is storm duration (h); *T_p_* is peak time (h); *T_L_* is lag time in hours; *Q_P_* is peak discharge (m^3^ s^−1^); and *A* is the drainage area (km^2^). The lag time is the time from the centroid of the excess rainfall to the hydrograph peak, while the time of concentration, which is denoted as *T_c_*, is defined as the time required for a particle of water to flow from the hydraulically most distant point in the watershed to the outlet or design point.

## 3. Results and Discussion

This section presents the rainfall analysis and the hydrology of the basin results due to the impact of CC on the watershed basin in high-stress regions.

### 3.1. Impact of the Recurrence Intervals on Rainfall Intensity

**Figure 4:**
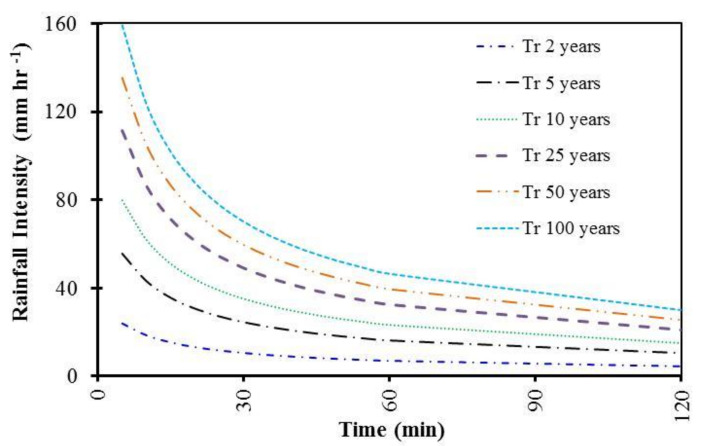
IDF curve for the study area.

Figure 4 presents the intensity duration frequency (IDF) curve for the study area at different storm times of 0, 30, 60, 90 and 120 min and recurrence intervals of 2, 5, 10, 25, 50 and 100 years, respectively. These charts can be used for the design of protection structures and drainage systems for different predictions of storm time and rainfall depths. 

### 3.2. Estimation of a Watershed Hydrograph

The WMS results were presented in Table 1 for the differences between 2, 5, 10, 25, 50, 100 and 200 years.

#### 3.2.1. The Peak Discharge Value 

**Figure 5:**
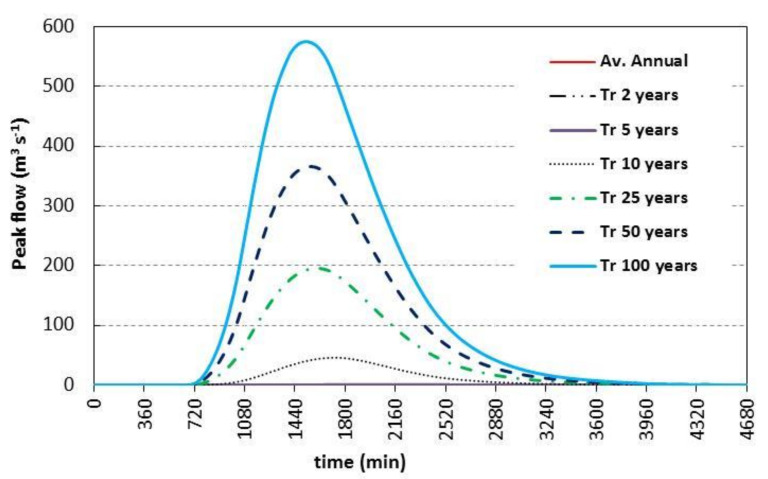
Runoff discharge for W. El Aawag watershed for different recurrence interval.

The results showed that the peak discharge reached 0, 0.44, 45.72, 195.45, 365.91 and 575.30 m^3^ s^−1^ at recurrence intervals of 2, 5, 10, 25, 50, and 100 years, with time to peak of 0, 2010, 1710, 1590, 1560 and 1530 min and precipitation of 15.20, 35.30, 50.60, 70.70, 85.90, and 101 mm, respectively, as shown in Figure 5. Moreover, the annual peak discharge reached 0 m^3^ s^−1^ at average annual precipitation of 13.37 mm; the 0 m^3^ s^−1^ value of runoff indicates that all precipitation goes as losses in the study area.

#### 3.2.2. The Flood Volume Value

The flood volume reached 0, 23,149.80, 2,896,241.40, 12,664,963.80 and 36,681,492.60 m^3^ at recurrence intervals of 2, 5, 10, 25, 50, and 100 years, respectively (see Figure 5). Additionally, the flood volume reached 0 at average annual precipitation of 13.37 mm, meaning all precipitation goes as losses, as presented in Table 2.

## 4. Discussion

Water resources for floods, droughts, severe summers, extreme heatwaves, mild cool weather occurrences, storms and other extreme weather events are all caused by CC [33,34,35]. Drought ranks top among CC-induced natural hazards in terms of the impact on the livelihood of the community [25,36,37,38]. Droughts are one of nature’s most harmful and destructive events. A drought is defined as a time when an area or region receives insufficient or below-normal precipitation. Reduced soil moisture, as well as surface and groundwater storages, are the results. A meteorological drought is defined as a scenario in which rainfall falls below 75% of the climatological normal in a certain area [39]. Increased warmth, water stress, the frequency of El Nino occurrences and the absence of available moisture in the sky all cause precipitation to decrease, resulting in fewer rainy days and more droughts, particularly in arid and semi-arid agro-ecologies [40,41]. Droughts have dramatically affected crop productivity and the quality of pastoral ecosystems in arid and semi-arid areas. Reduced and uneven rainfall during the monsoon season causes crop failure in early-season droughts and production losses in mid-season and late-season droughts in arid and semi-arid regions, according to reports [42,43].

**Figure 6:**
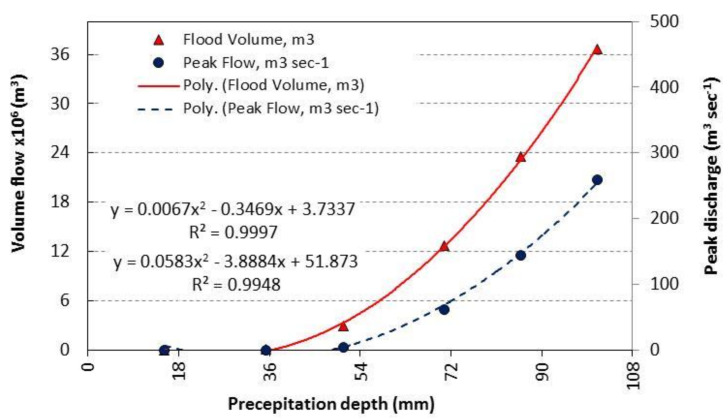
Relation between the projection precipitation depths and watershed hydrograph.

From the analysis of rainfall at El Tur stations from 1995 to 2021 using Hyfran-plus software, the results showed the depths of predicted precipitation as 15.20, 35.30, 50.60, 70.70, 85.90 and 101 mm, respectively, at recurrence intervals of 2, 5, 10, 25, 50 and 100 years with an annual depth of 13.37mm. The rainfall intensity was also estimated at different duration of the storms. Moreover, the WMS results showed that the flood volume reached 0, 23149.80, 2,896,241.40, 12,664,963.80 and 36,681,492.60 m^3^ and the peak discharge reached 0, 0.44, 45.72, 195.45, 365.91 and 575.30 m^3^ s^−1^ at the recurrence intervals of 2, 5, 10, 25, 50 and 100 years, respectively. The average annual yearly discharge and volume reached 0 m^3^ s^−1^ and 0 m^3^, respectively. Figure 6 showed the relation between the predicted precipitation depths and hydrograph volume and discharge in the study area. Furthermore, Equation (6) is useful to get the predicted hydrograph volume while the Equation (7) estimates the predicted discharge in the study area at diffrent depths of precipitation that is will change due to CC for drought.

(6) $$y=0.0067x^{2}-0.3469x+3.7337$$

(7) $$y=0.0583x^{2}-3.8884x+51.873$$

Modrick et al. (2015) [44] showed that the increasing in FF occurrences for the mountainous in small basins of Southern California due to projected CC between 30% and 40%. Overall, a decrease in the total number of precipitation events was found, although with increased precipitation intensity, increased event duration and higher soil saturation conditions for the 21st century. This combination could signify more hazardous conditions, with fewer precipitation events but higher rainfall intensity and over soils with higher initial soil moisture saturation, leading to the more frequent occurrence of FF. Esposito et al. (2018) [45] showed that increased frequency of FF events occurred on the coastline of the Campi Flegrei Volcanic Area, Italy. The variation in FF frequency is likely not related to urbanization changes, as no increase in the urban area occurred after the year 2000. The observed increase of FF events in recent years (2000–2014) can be reasonably ascribed to variations in the rainfall regime. Ragettli et al. (2021) [46] studied the impact of CC on summer flood frequencies in two mountainous catchments in China and Switzerland. The study recalibrated the weather generator with the climate statistics for 2021–2050 which it obtained from ensembles of bias-corrected regional climate models. Across all assessed return periods (10–100 years) and two emission scenarios, nearly all model chains indicate an intensification of flood extremes. According to the ensemble averages, the potential flood magnitudes increase by more than 30% in both catchments.

## 5. Conclusions

Water resources in desert regions are highly sensitive to CC, water demand and FF. The current study was developed in the desert of El-Qaa plain in Southwestern Sinai, Egypt, using the watershed modeling system software (WMS) to study the influence of CC considering different recurrence intervals of 2, 5, 10, 25, 50, 100 and 200 years. The results showed that the flood volume reached 0, 23,149.80, 2,896,241.40, 12,664,963.80 and 36,681,492.60 m^3^ while the peak discharge reached 0, 0.44, 45.72, 195.45, 365.91 and 575.30 m^3^ s^−1^ at recurrence intervals of 2, 5, 10, 25, 50 and 100 years, respectively. The annual volume and discharge reached 0 m^3^ and m^3^/s, respectively.

The prediction results of FF hazards for figures and equations at different recurrence intervals are useful for the decision-makers and engineers to consider in the future planning, development and design of rainwater harvesting and protection structures to keep the people living and safe in this desert area. Moreover, the study recommends estimating the sediment transport volume due to the influence of recurrence intervals under CC in desert regions around the world.

## Figures and Tables

**Table 1:** Historical climate data form weather of El Tur station.

Year	T_min_	T_max_	T_aver_	V	P_T_	R_A_	P_max_	P_aver_
1995	22.9	27.7	18.1	24.8	0	1	0	0
1996	23.2	28.2	18.5	24.5	13.46	2	12.95	6.73
1997	22.9	28	17.9	22.4	1.02	2	1.02	0.51
1998	23.7	28.4	19.1	23	3.05	1	2.03	3.05
2000	22	27.2	17	24.1	11.44	5	5.08	2.288
2001	23.3	28.2	18.1	25.7	11.94	1	11.94	11.94
2003	23.3	28.2	18.3	24.8	3.05	1	2.03	3.05
2004	22.9	27.7	18.2	25	0	1	0	0
2006	23.4	28.2	18.6	24.4	0.76	1	0.76	0.76
2007	23.6	28.6	18.8	23.6	0	1	0	0
2008	23.7	28.7	18.8	23.5	2.54	1	2.03	2.54
2009	23.7	28.9	18.6	22.6	0	1	0	0
2010	24.9	30	20	22.3	23.37	2	13.97	11.685
2011	23.5	28.1	18.7	24.1	2.03	2	2.03	1.015
2015	23.9	28.9	19	26.2	77.72	2	70.1	38.86
2017	23.5	28.5	18.6	19.5	70.61	2	70.1	35.305
2019	23.8	28.9	19	22.3	3.3	3	2.03	1.1
2021	24.3	28.9	19.4	26.2	273.31	4	199.9	68.3275

**Table 2:** Runoff discharge for W. El Aawag watershed.

Recurrence Intervals (Y)	Average Annual	2	5	10	25	50	100
Depth (mm)	13.37	15.3	35.30	50.60	70.70	85.90	101
**Time (min)**	**Flow (m^3^ s^−1^)**						
0	0.00	0.00	0.00	0.00	0.00	0.00	0.00
180	0.00	0.00	0.00	0.00	0.00	0.00	0.00
360	0.00	0.00	0.00	0.00	0.00	0.00	0.00
540	0.00	0.00	0.00	0.00	0.00	0.00	0.00
720	0.00	0.00	0.00	0.00	0.47	1.39	2.71
900	0.00	0.00	0.00	1.39	17.05	39.42	68.99
1080	0.00	0.00	0.00	7.84	66.92	145.47	246.98
1260	0.00	0.00	0.00	21.07	136.34	278.63	457.76
1440	0.00	0.00	0.03	35.11	183.02	354.73	565.87
1620	0.00	0.00	0.15	44.14	195.25	361.82	562.28
1800	0.00	0.00	0.34	44.85	173.38	307.78	465.77
1980	0.00	0.00	0.44	37.35	134.35	233.32	348.32
2160	0.00	0.00	0.40	27.17	95.09	163.81	243.31
2340	0.00	0.00	0.30	17.98	62.41	107.21	158.96
2520	0.00	0.00	0.18	11.35	39.50	67.90	100.71
2700	0.00	0.00	0.12	7.28	25.30	43.47	64.45
2880	0.00	0.00	0.08	4.67	16.22	27.88	41.36
3060	0.00	0.00	0.05	2.98	10.37	17.83	26.44
3240	0.00	0.00	0.03	1.90	6.63	11.39	16.89
3420	0.00	0.00	0.02	1.22	4.29	7.40	11.01
3600	0.00	0.00	0.01	0.80	2.80	4.83	7.18
3780	0.00	0.00	0.01	0.51	1.75	2.97	4.38
3960	0.00	0.00	0.01	0.31	0.94	1.52	2.15
4140	0.00	0.00	0.00	0.16	0.46	0.72	1.00
4320	0.00	0.00	0.00	0.07	0.19	0.30	0.41
4500	0.00	0.00	0.00	0.02	0.05	0.08	0.11
4680	0.00	0.00	0.00	0.00	0.00	0.00	0.00
4860	0.00	0.00	0.00	0.00	0.00	0.00	0.00
5040	0.00	0.00	0.00	0.00	0.00	0.00	0.00
5220	0.00	0.00	0.00	0.00	0.00	0.00	0.00
5400	0.00	0.00	0.00	0.00	0.00	0.00	0.00

## Data Availability

Not applicable.
